# Psychiatric and neurological disorders are associated with bullous pemphigoid – a nationwide Finnish Care Register study

**DOI:** 10.1038/srep37125

**Published:** 2016-11-15

**Authors:** Anna-Kaisa Försti, Jari Jokelainen, Hanna Ansakorpi, Allan Seppänen, Kari Majamaa, Markku Timonen, Kaisa Tasanen

**Affiliations:** 1 PEDEGO Research Unit, University of Oulu; Department of Dermatology and Medical Research Center Oulu, Oulu University Hospital, P.O. Box 20, 90029 Oulu University Hospital, Oulu, Finland; 2 Unit of General Practice, P.O. Box 10, 90029 Oulu University Hospital, Oulu, Finland; 3 Center for Life Course Epidemiology and Systems Medicine, P.O. Box 5000, 90014, University of Oulu, Oulu, Finland; 4 Research Unit of Clinical Neuroscience, University of Oulu; Department of Neurology and Medical Research Center Oulu, Oulu University Hospital, P.O. Box 5000, 90014, University of Oulu, Oulu, Finland; 5 Psychoses and Forensic Psychiatry, Helsinki University Hospital, Vanha Valtatie 198, 04500, Kellokoski, Finland and Vanha Vaasa Hospital, Vierinkiventie 1, 65380 Vaasa, Finland

## Abstract

Bullous pemphigoid (BP) is an autoimmune blistering skin disease with increasing incidence. BP is associated with neurological disorders, but it has not been established, what subtypes of dementia and stroke are associated with BP, and what is the temporal relation between these diseases. Also, the association between BP and psychiatric disorders is controversial. We conducted a retrospective nationwide study, using the Finnish Care Register for Health Care diagnoses between 1987 and 2013. The study population of 4524 BP patients were compared with 66138 patients with basocellular carcinoma (BCC), neurological and psychiatric comorbid disorders were evaluated for both groups, and associations were estimated by Cox regression and logistic regression analyses. The strongest risk of developing BP was found after diagnosis of multiple sclerosis (MS) (OR=5.9, 95% CI 3.9–8.5). Among psychiatric diseases, the corresponding risk was strongest in schizophrenia (OR=2.7, 95% CI 2.0–3.5), and as a novel finding, also personality disorders (OR=2.2, 95% CI 1.3–3.3) preceded BP. In conclusion, many psychiatric disorders, especially schizophrenia, carry heightened risk for BP. Furthermore, several neurological diseases which cause central nervous system inflammation or degeneration were related to BP, and the association was strongest between MS and BP.

Bullous pemphigoid (BP) is an autoimmune skin disease with incidence varying between 0.25 and 4.28/100.000 per year^1^,^2^,^3^. The age-adjusted incidence of BP has been reported to be on the rise^1^,^2^,^3^. BP typically presents with severe itching and blistering of the skin, but in up to 20% of patients blisters are completely absent and only excoriations or eczematous, urticated lesions are observed^4^. BP typically affects elderly people, has many comorbidities and carries an elevated risk of death compared with the general population of the same age^1^,^2^,^3^,^4^. The pathogenesis of BP is characterized by autoantibodies directed against the hemidesmosomal components between the basal keratinocytes of the epidermis and the extracellular matrix of the dermis, resulting in subepidermal blister formation. The main autoantigen is bullous pemphigoid antigen 180 (BP180, also known as BPAG 2 or collagen XVII), though BP patients may also have autoantibodies against bullous pemphigoid antigen 230 (BP230 or BPAG1), another hemidesmosomal protein^4^.

Several studies have established an association between BP and neurological disorders, especially stroke, dementia and Parkinson’s disease^5^,^6^,^7^,^8^. The mechanism behind this association has been suggested to be an autoinflammatory reaction against BP180 or the neuronal isoform of BP230 in the human brain^9^,^10^. However, previous studies did not specify the types of dementia or cerebrovascular stroke. Neurological disorders precede BP^5^,^6^, but it is unclear, whether the risk of developing neurological disorders is elevated after a diagnosis of BP.

To date, only a few studies have examined the epidemiological association between BP and psychiatric conditions. It has been reported that BP patients are more likely than the general population to have schizophrenia before their BP diagnosis^7^, and unipolar and bipolar disorders are also associated with BP^11^. On the contrary, another study found no connection between BP and depression^12^ and none of these studies have reported whether the risk for psychiatric disorders is elevated after the diagnosis of BP.

In order to clarify which neurological and psychiatric disorders are associated with BP and their temporal relationships, we conducted a nationwide, retrospective study using the Finnish Care Register for Health Care with one of the largest BP cohorts to date. Patients with comprehensive register data of diagnoses, comorbidities and dates of death were available thanks to highly reliable Finnish registries^13^.

## Results

### Characteristics of BP patients and controls

We found 4524 patients with BP (2719 [approx. 60%] women) and 66138 patients with BCC (36240 [approx. 55%] women) among individuals who were aged over 40 years and who had been treated in Finnish hospitals between 1987 and 2013. The mean ± standard deviation age at the time of diagnosis was 77 ± 11 years for BP and 73 ± 12 years for BCC. The crude incidence of BP in Finland was 2.5/100,000 per year and using the European standard population as a reference, the age-adjusted incidence was 1.8/100,000 per year.

The most common neurological comorbidities in BP patients were cerebral infarction (n = 859), ‘other or unspecified dementia’ (n = 607), and Alzheimer’s disease (n = 617) and among psychiatric (primary) diagnoses, depression (n = 259) (Table 1).

### Neurological and psychiatric diseases and the risk of BP

Several central nervous system (CNS), but not peripheral nervous system diseases were associated with BP. Patients with multiple sclerosis (MS) had 5.9-fold higher risk of developing BP later in life compared with controls (Table 2). In comparison with controls, dementias were also strongly associated with heightened risk of subsequent BP: ‘other or unspecified dementia’ with 3.8-fold, vascular dementia with 3.6-fold and Alzheimer’s disease with 2.6-fold. The risk for subsequent BP was 2.1-fold higher in patients with subarachnoid hemorrhage, 2.6-fold higher in intracerebral hemorrhage and 1.8-fold higher in cerebral infarction than in controls. Among psychiatric diseases, especially schizophrenia (2.7-fold increased risk), schizotypal and delusional disorders (2.1-fold increased risk) and personality disorders (2.2-fold increased risk) preceded BP significantly more often than BCC (Table 2).

Compared with controls, BP patients had a significantly higher risk of neurological and psychiatric disorders following the diagnosis of BP, the risk of epilepsy was 2.0-fold higher, schizotypal and delusional disorders, 1.8-fold, and dementias 1.2–1.7-fold. The risk for viral meningitis was 2.3-fold and that for viral encephalitis 2.0-fold higher (Table 2).

The mean time between diagnosis of dementia and that of BP was three years while MS patients developed BP approximately 12 years after MS diagnosis. Psychiatric diagnoses occurred between approximately 7 and 11 years before BP (Table 2).

## Discussion

Our comprehensive, nationwide, registry-based study showed that several psychiatric disorders preceded BP. These disorders included schizophrenia, schizotypal and delusional disorders, bipolar affective disorder, major depressive disorder, personality disorders, and ‘neurotic, stress-related and somatoform disorders’. In previous studies, psychiatric comorbidity in BP has been controversial^7^,^11^,^12^ and the association between BP and schizophrenia has emerged only in the study by Chen and coworkers (2011), which found similar elevation of risk as did our study. Our results support those of Bastuji-Garin and colleagues^11^, which showed an association of BP with bipolar affective disorder and major depressive disorder.

Our finding of an association between BP and dementias are in line with previous reports, particularly with the pioneering work of Langan and colleagues^6^, which showed that diagnosis of dementia elevates the risk for BP. However our study is the first to classify BP-associated dementias into specific diagnostic categories, and the first to demonstrate that the risk of developing BP was nearly 3-fold after a diagnosis of Alzheimer’s disease, and nearly 4-fold higher following a diagnosis of vascular dementia, as well as one of ‘other or unspecified dementia’.

While previous studies have used an undefined category of stroke, we divided cerebrovascular disorders into subarachnoid hemorrhages, intracerebral hemorrhages, and cerebral infarctions. We found that each, despite different pathophysiology, carried an approximately 2-fold elevated risk for subsequent BP compared with controls. Among neurological disorders the strongest association, however, was found between MS and BP. In a large cohort of MS patients, the risk of developing a subsequent BP was nearly 7-fold^14^. Our novel finding was that BP occurred on average 12 years earlier in MS patients than in BP patients in general.

Regarding diagnoses following a BP diagnosis, we showed that the risk of developing epilepsy was increased 2-fold whereas the risk for incident dementias and cerebrovascular diseases was elevated, but not to the same extend as the risk for epilepsy. The previous studies, except the ones considering motor neuron diseases^8^ and strokes^15^, have most probably missed this connection because of small study populations or short follow-up time. Still, the 2-fold increased risk of viral meningitis or viral encephalitis following a diagnosis of BP was unexpected. Although the small number of cases can bias this result, one possible explanation for true causality could be the common practice of treating BP with immunosuppressive drugs.

The highest risk of developing BP in our study was in MS, a well-known autoimmune disease of the CNS. Besides MS, evidence has accumulated that neuroinflammation also plays a role in various of the other neurological and psychiatric diseases that showed an increased risk of BP in this study. Neuroinflammatory mechanisms have been recognized in neurodegenerative diseases such as Alzheimer’s disease^16^ and Parkinson’s disease^17^ as well as in schizophrenia^18^, but also in diseases with a more acute clinical phenotype e.g. cerebral infarction^19^. In addition, depression^20^ and, recently, bipolar and stress-related disorders^21^,^22^ have been suggested to have neuroinflammatory changes as pathophysiological factors. In neuroinflammation, different disease-specific pathomechanisms either peripherally or centrally, trigger the activation of glia, which in turn leads to the production of inflammatory mediators^20^.

Autoimmunity against BP180 is recognized as a crucial event in the pathogenesis of BP, but it is completely unknown what triggers the increase in autoantibodies in elderly people^4^,^23^. BP180 is strongly expressed in the cortex and hippocampus, regions of the brain that are predilection areas for e.g. Alzheimer’s disease-related lesions^24^. In addition, the neuronal isoforms of BP230 are widely expressed in the human central and peripheral nervous systems^24^,^25^. This, and BP’s association of several neurological and psychiatric diseases, has led to a hypothesis that neuroinflammation could lead to a cross-reactive immune response between neural and cutaneous antigens^6^,^9^,^10^.

So far only a handful of studies have analyzed the association between BP and neurological disorders at the molecular level by measuring IgG reactivity against BP autoantigens in the sera of patients with neurological diseases^26^,^27^,^28^. A recent study failed to detect autoantibodies against BP180 or BP230 in patients with Parkinson’s disease (n = 125) and MS (n = 50). However, the mean age of patients with Parkinson’s disease was 63 years and that of MS patients only 33 years, which may explain this study’s negative results^27^. In contrast, another very recent study found that nine out of 24 patients with Parkinson’s disease and six out of 26 patients with various dementia types had antibodies against BP180^28^. In this study, the age of the patients was not reported. The association between BP autoantibodies and dementia has also been shown in a French study of 69 dementia patients and 69 controls (aged over 69 years)^26^ in which BP180 autoantibodies were detected in 7% of subjects with dementia (Mini-Mental State Examination, [MMSE] score ≤24), but not in controls (MMSE score >24). Using ELISA assays, our research group has recently analyzed the IgG reactivity to BP autoantigens in the sera of 115 patients with Alzheimer’s disease and 40 neurologically healthy controls. Autoantibodies against the immunodominant NC16A domain of BP180 were found in 19% of Alzheimer’s disease patients, but only 2% of controls had positive results. The levels of BP180 autoantibodies correlated with the level of cognitive decline^29^.

Another factor behind the association between neurological and psychiatric disorders and BP could be the medication used for these diseases. Chronic use of both spironolactone and phenothiazine antipsychotics has been demonstrated to be associated with BP^11^. For now, the role of drugs serving as triggers for BP is poorly understood. The genetic background and HLA (human leukocyte antigen) -association in BP seems to be of less importance than in another autoimmune blistering skin disease, pemphigus^30^. Still, association between BP and HLA-DQB1*0301 antigen has been reported among Caucasian patients^31^. There is also a possibility that unknown changes in the immune systems of elderly people lead to the loss of immunotolerance against BP180/BP230 separately in the skin and brain.

Ours is one of the largest studies of BP, neurological and psychiatric diseases, and covers a long study period. Even so, the number of cases of personality disorder and bipolar affective disorder was relatively small and consequently further studies are required to confirm our findings. One limitation of the data set we examined was that data for outpatient visits were not available before 1998. A major limitation in the use of administrative registry data is that direct validation of diagnoses is not possible. However, studies assessing the internal validity of Finnish registries and comparing registry information with patient records have confirmed that the coverage and accuracy of Finnish registry data are well-suited for epidemiological research^13^.

We conclude that the association between BP and schizotypal and delusional disorders, dementias, epilepsy, and cerebrovascular diseases was bidirectional, but the risk of developing BP was higher after the corresponding neurological or psychiatric diagnosis than vice versa. However, the risk of incident BP was highest in patients with MS, and what is more, these patients were remarkably younger at the time of BP diagnosis than BP patients in general. Clinicians who treat neurological or psychiatric patients should take into account the possibility of BP: to examine the skin carefully, to screen for pruritus and to remember that pruritus may also manifest as anxiety. It also has to be remembered that in many BP cases, pruritus can precede blistering of the skin by several months, disturb sleeping, thus strongly affecting the individual’s cognition as well. In future, large prospective studies with well-characterized cohorts of neurological and psychiatric patients are required to understand in more detail the association of BP with neuroinflammation. It is also important to address the molecular mechanism leading to the breakage of immunological tolerance against BP autoantigens in the human brain.

## Methods

### Populations and databases

This was a retrospective database study of all BP cases diagnosed in every Finnish hospital between 1^st^ Jan 1987 and 31^st^ Dec 2013. The patient records were obtained from the statutory Finnish Care Register for Health Care (former name the Finnish Hospital Discharge Register) maintained by the National Institute of Health and Welfare, and selected by diagnoses based on the International Classification of Diseases (ICD 9 codes 6945 A and 6945B, and ICD 10 code L12.0). The Care Register covers all hospitals administered by local authorities, municipal federations and central government as well as the largest private hospitals. Each record contains the identification numbers of the patient and hospital, primary and subsidiary diagnoses, and duration of hospital stay. The Care Register also covers outpatient visits from 1998 onwards. In Finland, ICD-9 was used between 1987 and 1995 and ICD-10 since 1996.

In this study, the control population was formed of patients having basocellular carcinoma (BCC) (ICD 9 codes 1730A-1739A and ICD 10 codes C44.01, C44.11, C44.21, C44.31, C44.41, C44.51, C44.61, C44.71, C44.81, C44.91) since it is a common diagnosis, affects people in advanced age as does BP, and is not inflammatory in origin. The neurological and psychiatric comorbid diagnoses, including both primary and subsidiary diagnoses (Table 1), were gathered for both BP patients and controls from the Care Register for Health Care. To ensure the validity of psychiatric diagnoses, we calculated the association between BP and psychiatric diseases using solely primary psychiatric diagnoses made in the specialized care setting. The dates of death were provided by the Population Register Centre. Only records of patients aged 40 years and above were included in the study since BP is rarely seen in younger individuals^1^,^2^,^32^.

### Ethical aspects and permissions

The data of the study population and controls were obtained without identification and patients were not contacted, which is why a statement of the ethical committee was not required. However, permission was obtained from the National Institute of Health and Welfare and from the Data Protection Ombudsman.

### Outcomes

The primary outcome in this study was association between BP and neurological and psychiatric disorders. The secondary outcome was to determine any temporal causality between BP and neurological and psychiatric diseases, i.e. which condition precedes the other. A third outcome was to calculate the incidence of BP in the whole of Finland within the specified 26-year time period.

### Statistical analyses

The characteristics of the study population were presented as proportions and means. The associations between BP and a history of neurological and psychiatric disorders were evaluated using a logistic regression model and presented with odds ratios (OR) and 95% confidence intervals (CI). The risk of developing neurological and psychiatric disorders during follow-up for BP or BCC were compared between patients and controls using the Cox proportional hazards regression model and presented with hazard ratios (HR) and 95% CI. The duration of follow-up was defined as the time from the date of diagnosis of BP or BCC to the date of diagnosis of neurologic or psychiatric disorder of interest. If the diagnosis of neurological or psychiatric disorder of interest did not appear, the follow-up ended at the date of death or at the date of last visit record. Both statistical models were adjusted for age at the time of diagnosis and for gender.

The crude incidence rate was calculated as the ratio of the number of new BP cases in one year to the number of individuals who were at risk. The age-standardized incidence was calculated using indirect method and the Revised European Standard Population 2013 (ESP2013) as a reference. The standard population of Finland between 1987 and 2013 for different age groups was provided by Statistics Finland (http://www.tilastokeskus.fi).

All Statistical analyses were performed using STATA (Data Analysis and Statistical Software, MP 13, StataCorp LP, College Station, TX 77845, USA) and the SAS software package (version 9.4, SAS Institute, Inc) and two sided P-values less than 0.05 were considered statistically significant.

## Additional Information

**How to cite this article**: Försti, A.-K. *et al*. Psychiatric and neurological disorders are associated with bullous pemphigoid – a nationwide Finnish Care Register study. *Sci. Rep.* **6**, 37125; doi: 10.1038/srep37125 (2016).

**Publisher’s note:** Springer Nature remains neutral with regard to jurisdictional claims in published maps and institutional affiliations.

## Figures and Tables

**Table 1:** The number of neurological and psychiatric diseases among patients with bullous pemphigoid (BP) and basocellular carcinoma (BCC) before and after the corresponding (BP/BCC) diagnosis.

	ICD-9/ICD-10	Number of cases among BP patients before/after	Number of cases among BCC patients before/after
**Central nervous system diseases**			
Alzheimer’s disease	331.0 A/F00, G30	343/274	1520/3867
Vascular dementia	437.8X, 437.8 A/F01	100/101	325/964
Other/unspecified dementia	331.2X, 290.0 A/F03, G31^b^	342/265	1092/2462
Parkinson’s disease	332.0 A/G20, F02.3	146/45	812/730
Multiple sclerosis (MS)	340.0 A/G35, F02.89*G35	35/1	112/30
Essential tremor	333.1 A/G25.0	18/8	240/150
Degenerative diseases of basal ganglia	333.0 A/G23	4/2	16/34
Narcolepsy and cataplexy	347.1 A/G47.4	0/0	4/3
Subarachnoid hemorrhage	430.1 A, 430.0 A, 430.9X/I60	33/6	236/186
Intracerebral hemorrhage	431, 432/I61,I62	100/64	517/862
Cerebral infarction	433.0 A, 433.1 A, 433.9 A, 434.0 A, 434.1 A, 434.9 A/I63	539/320	4080/4208
Epilepsy	345/G40^c^	148/77	981/715
Migraine	346.0 A, 346.1 A, 346.9X/G43	22/9	432/177
Viral encephalitis	323.8B, 053.1 A, 054.3B, 052.0 A, 063.2 A, 056.0 A, 055.0 A, 049.9X, 064.0 A, 063.8X, 063.9X, 062.8X, 062.9X, 139.0 A/A84, A85, A86, B01.1+, A83.8, A83.9, G05.1*, B05.0+, B02.0, B00.4	5/10	92/84
Viral meningitis	047.0 A, 047.1 A, 047.8X, 047.9X, 320.0 A, 054.3 A, 053.0 A, 049.8X, 049.9X/A87, G02.0*, G03.9, B05.1+, B02.0, B02.1, B00.4	6/8	89/66
Bacterial meningoencephalitis	323.8 A, 036.1 A, 036.1B, 013.8 A, 013.8B, 137.1X/G04.2, G05.0*	0/0	8/4
Bacterial meningitis	320, 027.0 A, 036.0 A, 104.8 A, 003.2 A, 094.2 A, 130.0 A, 013.0 A, 013.0B, 326.9X/G00, G01*	3/2	35/17
**Peripheral nervous system diseases**			
Dystonia	333.6 A/G24^d^	11/1	119/59
Primary disorders of muscles and other myopathies	359^e^/G71, G72^f^	22/26	185/308
Cranial nerve disorders	350, 352/G50, G51, G52	43/12	684/317
Motor neuron disease (ALS)	335.2 A, 335.2B, 335.2 C/G12.2	3/4	16/57
Polyneuropathy	357.0 A, 356, 357.8X, 357.9X/G61, G62.8, G62.9, G60, G64	46/13	528/350
Mitochondrial myopathy	-^g^/G71.3, G32.8*	1/0	2/5
**Psychiatric diseases**^a^			
Schizotypal and delusional disorders (including schizoaffective disorders)	297.1 A, 297.3 A, 298.8 A, 298.9X/F22–29	81/33	584/316
Schizophrenia	295/F20, F21	53/5	343/72
Manic episode	-^g^/F30	2/2	36/28
Bipolar affective disorder	296.2, 296.3, 296.4, 296.7 A/F31	19/1	195/66
Major depressive disorder	296.1 A, 296.8 A, 300.4 A/F32, F33, F34.1	200/59	2095/970
Neurotic, stress-related and somatoform disorders	300^h^, 309.0 A, 309.2 C, 309.2D, 309.2E, 309.3 A, 309.4 A, 309.8 A, 309.8X, 309.9X/F40–45, F48	95/37	1226/564
Personality disorder	301/F60–69	21/2	185/31

Abbreviations: ICD, International Classification of Diseases; BP, bullous pemphigoid; BCC, basocellulare carcinoma. ^a^Primary diagnoses, ^b^Excluding G31.2, ^c^Excluding G40.51, G40.52, ^d^Excluding G24.0#, ^e^Excluding 359.4 A,359.4B, ^f^Excluding G71.10#, G71.3, G72.0#, G72.1, G72.2, ^g^Not corresponding diagnosis in ICD-9, ^h^Excluding 300.4 A.

**Table 2:** Odds ratios (OR) in patients with neurological or psychiatric disease for bullous pemphigoid (BP) and mean time to BP diagnosis.

	OR (95% CI)^a^	Mean time to BP, years (std.dev)	HR (95% CI)^*a*^	Mean time to comorbid disease, years (std.dev)
**Central nervous system diseases**				
Alzheimer’s disease	2.64 (2.33–2.99)	3.1 (2.5)	1.24 (1.09–1.40)	4.0 (4.0)
Vascular dementia	3.58 (2.84–4.49)	3,0 (2.7)	1.67 (1.36–2.05)	3.5 (3.8)
Other/unspecified dementia	3.84 (3.37–4.37)	3.1 (3.0)	1.75 (1.54–1.99)	3.7 (4.1)
Parkinson’s disease	2.40 (2.00–2.87)	4.6 (4.0)	1.13 (0.83–1.52)	2.2 (2.8)
Multiple sclerosis (MS)	5.87 (3.93–8.53)	12.2 (6.0)	0.67 (0.09–4.95)	1.6 (^c^)
Subarachnoid hemorrhage	2.05 (1.40–2.92)	8.5 (6.0)	0.58 (0.26–1.31)	3.4 (3.2)
Intracerebral hemorrhage	2.62 (2.10–3.24)	6.8 (5.6)	1.30 (1.01–1.68)	3.5 (3.4)
Cerebral infarction	1.79 (1.62–1.97)	5.5 (4.9)	1.30 (1.16–1.46)	4.1 (4.0)
Epilepsy	2.33 (1.95–2.77)	7.9 (6.8)	1.97 (1.55–2.49)	3.8 (3.7)
Viral encephalitis	0.80 (0.28–1.77)	8.5 (5.6)	2.04 (1.05–3.93)	2.8 (3.4)
Viral meningitis	1.06 (0.41–2.24)	7.3 (6.4)	2.28 (1.09–4.77)	3.6 (3.9)
**Psychiatric diseases**^b^				
Schizotypal and delusional disorders	2.06 (1.62–2.60)	7.5 (6.2)	1.82 (1.27–2.61)	3.7 (4.5)
Schizophrenia	2.65 (1.95–3.51)	11.7 (9.0)	1.36 (0.55–3.37)	3.3 (4.6)
Bipolar affective disorder	1.70 (1.02–2.66)	9.4 (6.8)	0.31 (0.04–2.23)	7.9 (^c^)
Major depressive disorder	1.51 (1.29–1.75)	7.1 (5.2)	1.14 (0.88–1.49)	3.7 (4.4)
Neurotic, stress-related and somatoform disorders	1.27 (1.02–1.57)	7.3 (5.8)	1.24 (0.89–1.74)	2.9 (3.0)
Personality disorder	2.17 (1.33–3.33)	11.2 (7.7)	1.25 (0.30–5.23)	6.8 (9.5)

Hazard ratios (HR) in patients with BP for neurological and psychiatric diseases and mean time to comorbid (neurological or psychiatric) diagnosis. Only diseases with statistically significant OR or HR included.

OR, odds ratio; std.dev, standard deviation; HR, hazard ratio. ^a^Adjusted for sex and age, ^b^Primary diagnoses, cIncludes only one case, std.dev cannot be calculated.
